# *catena*-Poly[[(18-crown-6-κ^6^ *O*)potassium]-μ-chlorido-[(1*H*-benzotriazol-1-ol-κ*N* ^3^)chloridoplatinum(II)]-μ-(benzotriazol-1-olato-κ^2^ *N* ^3^:*O*)]

**DOI:** 10.1107/S1600536810015898

**Published:** 2010-05-08

**Authors:** B. Ravindran Durai Nayagam, Samuel Robinson Jebas, J. Shakina, R. Murugesan, Dieter Schollmeyer

**Affiliations:** a Department of Chemistry, Popes College, Sawyerpuram 628 251, India; b Department of Physics, Sethupathy Government Arts College, Ramanathapuram 623 502, Tamilnadu, India; c Department of Chemistry, Sarah Tucker College, Tirunelveli 627 007, Tamilnadu, India; d Department of Chemistry, T.D.M.N.S. College, T. Kallikulam, Tamilnadu, India; e Institut für Organische Chemie, Universität Mainz, Duesbergweg 10-14, 55099 Mainz, Germany

## Abstract

In the structure of the title compound, [KPt(C_6_H_4_N_3_O)Cl_2_(C_6_H_5_N_3_O)(C_12_H_24_O_6_)], the Pt^II^ atom is in a distorted square-planar geometry. The crystal structure is consolidated by O—H⋯O hydrogen bonds. The measured crystal was a non-merohedral twin with four components.

## Related literature

For related literature, see Anderson *et al.* (1963 ▶). For related structures, see Bosch *et al.* (1983 ▶).
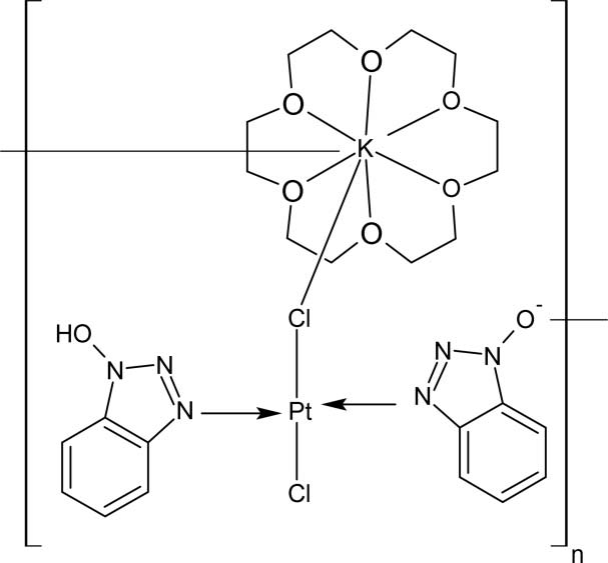


## Experimental

### 

#### Crystal data

[KPt(C_6_H_4_N_3_O)Cl_2_(C_6_H_5_N_3_O)(C_12_H_24_O_6_)]
*M* *_r_* = 838.65
Monoclinic, 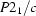

*a* = 14.2727 (13) Å
*b* = 10.9821 (10) Å
*c* = 20.0716 (18) Å
β = 99.249 (3)°
*V* = 3105.2 (5) Å^3^
*Z* = 4
Mo *K*α radiation
μ = 4.88 mm^−1^
*T* = 173 K
0.17 × 0.10 × 0.08 mm

#### Data collection

Bruker SMART APEXII CCD diffractometer
Absorption correction: multi-scan (*TWINABS*; Sheldrick, 1999 ▶) *T*_min_ = 0.494, *T*_max_ = 0.746
10775 measured reflections
10775 independent reflections
9006 reflections with *I* > 2σ(*I*)

#### Refinement

*R*[*F* ^2^ > 2σ(*F* ^2^)] = 0.063
*wR*(*F* ^2^) = 0.178
*S* = 1.07
10775 reflections
382 parameters
H-atom parameters constrained
Δρ_max_ = 1.77 e Å^−3^
Δρ_min_ = −1.78 e Å^−3^

### 

Data collection: *APEX2* (Bruker, 2008 ▶); cell refinement: *SAINT-Plus* (Bruker, 2008 ▶); data reduction: *SAINT-Plus*; program(s) used to solve structure: *SHELXS97* (Sheldrick, 2008 ▶); program(s) used to refine structure: *SHELXL97* (Sheldrick, 2008 ▶); molecular graphics: *SHELXTL* (Sheldrick, 2008 ▶); software used to prepare material for publication: *SHELXTL* and *PLATON* (Spek, 2009 ▶).

## Supplementary Material

Crystal structure: contains datablocks global, I. DOI: 10.1107/S1600536810015898/bt5255sup1.cif

Structure factors: contains datablocks I. DOI: 10.1107/S1600536810015898/bt5255Isup2.hkl

Additional supplementary materials:  crystallographic information; 3D view; checkCIF report

## Figures and Tables

**Table 1:** Hydrogen-bond geometry (Å, °)

*D*—H⋯*A*	*D*—H	H⋯*A*	*D*⋯*A*	*D*—H⋯*A*
O20—H20⋯O10^i^	0.84	1.76	2.466 (12)	141

Symmetry code: (i) 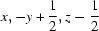.

**Fig. 1.**
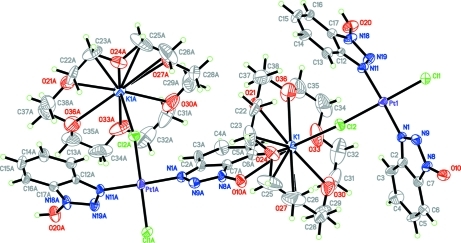
The molecular structure of the title compound, showing 50% probability displacement ellipsoids and the atom numbering scheme.

## supplementary crystallographic information

### Comment

For related literature, see  Anderson *et al.* (1963) and for related structures see Bosch *et al.* (1983).

The metal platinum exhibits the distorted square planar coordination geometry. The geometry is completed by two chorine atoms and two nitrogen atoms from the 1-oxobenzotriazole ligands. Distortion in the square-planar Pt coordination sphere is signaled by angles about Pt ranging from 88.9 (3) Å to 91.2 (3) Å. The K ion is coordinated by six oxygen atoms of the crown ether and also by one terminal Cl atom attached to the metal platinum and one O atom from the 1-oxobenzotriazole ligand forming a hexagonal pyramid. The potassium ion lies in the plane of the 18-crown 6-molecule, with mean K—O distance of 2.8 Å.

The crystal structure is consolidated by O—H···O hydrogen bonds.

### Experimental

A mixture of potassium tetrachloroplatinate(II) (0.0208 g, 0.05 mmol) and 1-hydroxybenzotriazole (0.0135 g, 0.1 mmol) 18-crown-6 ether (0.013 g 0.05 mmol) in acetone (15 ml) was heated at 313 K with stirring for 30 min. The yellow colour compound formed was filtered off, and dried. The compound was then dissolved in dichloromethane and kept at 278 K. Pale yellow crystals are formed after one month on slow evaporation.

### Refinement

All   H atoms were positioned geometrically  with C—H=0.95 Å (aromatic) C—H=0.98Å (methylene), O—H= 0.84Å and refined using a riding model with, *U*_iso_(H)=1.2U_equ_ (C) and 1.5U_equ_(O). The measured crystal was a non-merohedral twin with four components  0.536 (1) 0.194 (2), 0.226 (1) and 0.044 (1).

### Crystal data

**Table:**

[KPt(C_6_H_4_N_3_O)Cl_2_(C_6_H_5_N_3_O)(C_12_H_24_O_6_)]	*F*(000) = 1656
*M**_r_* = 838.65	*D*_x_ = 1.794 Mg m^−^^3^
Monoclinic, *P*2_1_/*c*	Mo *K*α radiation, λ = 0.71069 Å
Hall symbol: -P 2ybc	Cell parameters from 8964 reflections
*a* = 14.2727 (13) Å	θ = 2.3–26.9°
*b* = 10.9821 (10) Å	µ = 4.88 mm^−^^1^
*c* = 20.0716 (18) Å	*T* = 173 K
β = 99.249 (3)°	Block, light yellow
*V* = 3105.2 (5) Å^3^	0.17 × 0.10 × 0.08 mm
*Z* = 4	

### Data collection

**Table:**

Bruker SMART CCD diffractometer	10775 independent reflections
Radiation source: sealed Tube	9006 reflections with *I* > 2σ(*I*)
graphite	*R*_int_ = 0.0000
CCD scan	θ_max_ = 28.0°, θ_min_ = 1.5°
Absorption correction: multi-scan (TWINABS; Sheldrick, 1999)	*h* = −18→18
*T*_min_ = 0.494, *T*_max_ = 0.746	*k* = 0→14
10775 measured reflections	*l* = 0→26

### Refinement

**Table:**

Refinement on *F*^2^	Primary atom site location: structure-invariant direct methods
Least-squares matrix: full	Secondary atom site location: difference Fourier map
*R*[*F*^2^ > 2σ(*F*^2^)] = 0.063	Hydrogen site location: inferred from neighbouring sites
*wR*(*F*^2^) = 0.178	H-atom parameters constrained
*S* = 1.07	*w* = 1/[σ^2^(*F*_o_^2^) + (0.0972*P*)^2^ + 18.2058*P*] where *P* = (*F*_o_^2^ + 2*F*_c_^2^)/3
10775 reflections	(Δ/σ)_max_ = 0.001
382 parameters	Δρ_max_ = 1.77 e Å^−^^3^
0 restraints	Δρ_min_ = −1.78 e Å^−^^3^

### Special details

**Table:**

Geometry. All esds (except the esd in the dihedral angle between two l.s. planes) are estimated using the full covariance matrix. The cell esds are taken into account individually in the estimation of esds in distances, angles and torsion angles; correlations between esds in cell parameters are only used when they are defined by crystal symmetry. An approximate (isotropic) treatment of cell esds is used for estimating esds involving l.s. planes.
Refinement. Refinement of *F*^2^ against ALL reflections. The weighted *R*-factor wR and goodness of fit *S* are based on *F*^2^, conventional *R*-factors *R* are based on *F*, with *F* set to zero for negative *F*^2^. The threshold expression of h *F*^2^ > σ(*F*^2^) is used only for calculating *R*-factors(gt) etc. and is not relevant to the choice of reflections for refinement. *R*-factors based on *F*^2^ are statistically about twice as large as those based on *F*, and *R*- factors based on ALL data will be even larger. Crystal was split into 4 fragments and refined using HKLF 5/BASF method'

### Fractional atomic coordinates and isotropic or equivalent isotropic displacement parameters (Å^2^)

**Table:**

	*x*	*y*	*z*	*U*_iso_*/*U*_eq_	
Pt1	0.26231 (3)	0.37872 (3)	0.527664 (17)	0.02221 (11)	
Cl1	0.2212 (2)	0.2066 (3)	0.58061 (15)	0.0375 (7)	
Cl2	0.3097 (2)	0.5510 (2)	0.47650 (14)	0.0352 (6)	
N1	0.2340 (6)	0.4823 (8)	0.6036 (4)	0.0229 (17)	
C2	0.1620 (8)	0.5710 (9)	0.5966 (5)	0.025 (2)	
C3	0.0955 (8)	0.6111 (11)	0.5421 (5)	0.033 (2)	
H3	0.0940	0.5768	0.4985	0.040*	
C4	0.0340 (8)	0.6989 (12)	0.5523 (7)	0.042 (3)	
H4	−0.0100	0.7293	0.5156	0.051*	
C5	0.0348 (10)	0.7488 (11)	0.6216 (7)	0.039 (3)	
H5	−0.0113	0.8079	0.6284	0.047*	
C6	0.0992 (9)	0.7126 (10)	0.6750 (6)	0.033 (2)	
H6	0.1018	0.7468	0.7188	0.039*	
C7	0.1626 (8)	0.6199 (10)	0.6609 (5)	0.026 (2)	
N8	0.2308 (7)	0.5555 (8)	0.7023 (4)	0.0261 (18)	
N9	0.2737 (7)	0.4742 (8)	0.6659 (4)	0.0281 (19)	
O10	0.2552 (6)	0.5648 (7)	0.7691 (4)	0.0341 (18)	
N11	0.2852 (7)	0.2812 (8)	0.4469 (4)	0.0256 (19)	
C12	0.3658 (7)	0.2703 (9)	0.4180 (5)	0.022 (2)	
C13	0.4600 (9)	0.3121 (10)	0.4392 (5)	0.029 (2)	
H13	0.4781	0.3549	0.4804	0.035*	
C14	0.5228 (9)	0.2866 (11)	0.3965 (6)	0.034 (3)	
H14	0.5870	0.3119	0.4081	0.040*	
C15	0.4941 (9)	0.2230 (12)	0.3349 (6)	0.038 (3)	
H15	0.5403	0.2078	0.3067	0.045*	
C16	0.4049 (9)	0.1832 (10)	0.3144 (6)	0.030 (2)	
H16	0.3864	0.1416	0.2728	0.036*	
C17	0.3423 (8)	0.2076 (9)	0.3586 (5)	0.028 (2)	
N18	0.2459 (7)	0.1814 (9)	0.3558 (4)	0.031 (2)	
N19	0.2129 (6)	0.2248 (8)	0.4094 (4)	0.0274 (19)	
O20	0.1871 (6)	0.1243 (9)	0.3072 (4)	0.0410 (19)	
H20	0.2188	0.0847	0.2828	0.062*	
O21	0.4161 (7)	0.6331 (9)	0.3022 (5)	0.047 (2)	
C22	0.4979 (11)	0.6571 (17)	0.3516 (7)	0.056 (4)	
H22A	0.4980	0.6022	0.3907	0.067*	
H22B	0.5561	0.6407	0.3320	0.067*	
C23	0.4981 (11)	0.7788 (17)	0.3731 (9)	0.060 (5)	
H23A	0.5588	0.7971	0.4028	0.073*	
H23B	0.4923	0.8336	0.3335	0.073*	
O24	0.4228 (7)	0.7993 (8)	0.4081 (5)	0.041 (2)	
C25	0.4190 (14)	0.9189 (16)	0.4377 (13)	0.084 (7)	
H25A	0.4796	0.9350	0.4680	0.100*	
H25B	0.4119	0.9808	0.4014	0.100*	
C26	0.3432 (19)	0.9305 (19)	0.4746 (11)	0.093 (7)	
H26A	0.3465	1.0111	0.4969	0.111*	
H26B	0.3490	0.8672	0.5101	0.111*	
O27	0.2539 (9)	0.9180 (9)	0.4314 (5)	0.058 (3)	
C28	0.1692 (19)	0.9269 (17)	0.4658 (11)	0.085 (7)	
H28A	0.1752	0.8691	0.5041	0.103*	
H28B	0.1639	1.0103	0.4835	0.103*	
C29	0.084 (2)	0.897 (2)	0.4162 (15)	0.108 (10)	
H29A	0.0261	0.9117	0.4364	0.129*	
H29B	0.0813	0.9504	0.3760	0.129*	
O30	0.0883 (8)	0.7821 (13)	0.3984 (6)	0.072 (4)	
C31	0.0019 (12)	0.740 (3)	0.3545 (10)	0.090 (8)	
H31A	−0.0530	0.7486	0.3787	0.108*	
H31B	−0.0100	0.7918	0.3137	0.108*	
C32	0.0092 (14)	0.613 (3)	0.3341 (10)	0.092 (8)	
H32A	0.0174	0.5602	0.3746	0.111*	
H32B	−0.0504	0.5886	0.3048	0.111*	
O33	0.0817 (8)	0.5961 (12)	0.3011 (6)	0.072 (4)	
C34	0.0933 (18)	0.468 (2)	0.2871 (12)	0.096 (8)	
H34A	0.0316	0.4331	0.2665	0.115*	
H34B	0.1158	0.4242	0.3297	0.115*	
C35	0.1637 (17)	0.4551 (19)	0.2400 (11)	0.092 (7)	
H35A	0.1704	0.3682	0.2285	0.110*	
H35B	0.1420	0.5006	0.1978	0.110*	
O36	0.2516 (10)	0.5015 (11)	0.2723 (6)	0.074 (4)	
C37	0.3247 (16)	0.4920 (14)	0.2324 (8)	0.072 (6)	
H37A	0.3163	0.5539	0.1960	0.086*	
H37B	0.3247	0.4102	0.2117	0.086*	
C38	0.4148 (16)	0.5133 (16)	0.2799 (8)	0.069 (5)	
H38A	0.4188	0.4571	0.3189	0.083*	
H38B	0.4699	0.4980	0.2568	0.083*	
K1	0.25264 (18)	0.7160 (2)	0.34906 (12)	0.0318 (5)	

### Atomic displacement parameters (Å^2^)

**Table:**

	*U*^11^	*U*^22^	*U*^33^	*U*^12^	*U*^13^	*U*^23^
Pt1	0.02707 (18)	0.02415 (16)	0.01630 (15)	0.0034 (2)	0.00617 (14)	−0.00098 (16)
Cl1	0.0513 (18)	0.0285 (13)	0.0382 (15)	0.0003 (12)	0.0242 (14)	0.0044 (12)
Cl2	0.0531 (17)	0.0260 (12)	0.0287 (13)	−0.0019 (12)	0.0135 (12)	0.0038 (11)
N1	0.026 (4)	0.025 (4)	0.018 (4)	0.005 (3)	0.004 (3)	0.004 (3)
C2	0.027 (5)	0.028 (5)	0.023 (5)	0.002 (4)	0.007 (4)	0.000 (4)
C3	0.043 (6)	0.035 (6)	0.022 (5)	0.014 (5)	0.005 (4)	0.000 (5)
C4	0.018 (5)	0.051 (8)	0.055 (8)	0.014 (5)	−0.003 (5)	0.009 (6)
C5	0.045 (7)	0.033 (6)	0.041 (7)	0.015 (5)	0.016 (6)	−0.005 (5)
C6	0.037 (6)	0.026 (5)	0.037 (6)	−0.001 (5)	0.013 (5)	0.000 (5)
C7	0.036 (6)	0.025 (4)	0.019 (4)	−0.002 (5)	0.005 (4)	−0.001 (4)
N8	0.036 (5)	0.030 (5)	0.013 (4)	0.004 (4)	0.005 (3)	−0.002 (3)
N9	0.037 (5)	0.024 (4)	0.023 (4)	0.007 (4)	0.004 (4)	0.007 (3)
O10	0.049 (5)	0.031 (4)	0.021 (4)	0.008 (4)	0.002 (3)	−0.002 (3)
N11	0.035 (5)	0.029 (4)	0.013 (4)	0.006 (4)	0.004 (3)	−0.003 (3)
C12	0.030 (5)	0.019 (4)	0.020 (4)	0.003 (4)	0.011 (4)	0.001 (4)
C13	0.037 (6)	0.031 (5)	0.018 (5)	−0.002 (5)	0.002 (5)	−0.002 (4)
C14	0.031 (6)	0.037 (6)	0.034 (6)	0.009 (5)	0.007 (5)	−0.001 (5)
C15	0.041 (7)	0.048 (7)	0.027 (6)	0.001 (6)	0.016 (5)	−0.006 (5)
C16	0.039 (6)	0.031 (5)	0.023 (5)	0.006 (5)	0.013 (5)	0.003 (4)
C17	0.029 (6)	0.023 (5)	0.033 (5)	0.001 (4)	0.013 (5)	0.001 (4)
N18	0.040 (6)	0.036 (5)	0.016 (4)	0.003 (4)	0.001 (4)	−0.013 (4)
N19	0.028 (5)	0.035 (5)	0.020 (4)	−0.002 (4)	0.002 (3)	−0.009 (4)
O20	0.043 (5)	0.049 (5)	0.028 (4)	0.010 (5)	−0.003 (3)	−0.011 (4)
O21	0.057 (6)	0.047 (5)	0.038 (4)	0.003 (5)	0.010 (4)	0.009 (4)
C22	0.051 (8)	0.094 (13)	0.028 (6)	0.019 (8)	0.023 (6)	0.009 (7)
C23	0.033 (7)	0.081 (12)	0.066 (10)	−0.005 (8)	0.004 (7)	0.035 (9)
O24	0.045 (5)	0.033 (4)	0.040 (5)	−0.004 (4)	−0.014 (4)	0.006 (4)
C25	0.067 (12)	0.041 (8)	0.127 (19)	−0.007 (8)	−0.032 (12)	−0.025 (10)
C26	0.13 (2)	0.058 (11)	0.084 (14)	0.005 (13)	−0.008 (15)	−0.035 (10)
O27	0.094 (9)	0.038 (5)	0.047 (6)	0.015 (5)	0.025 (6)	−0.002 (4)
C28	0.14 (2)	0.049 (9)	0.084 (14)	−0.011 (11)	0.081 (15)	−0.017 (9)
C29	0.13 (2)	0.090 (17)	0.13 (2)	0.052 (16)	0.086 (19)	0.021 (16)
O30	0.052 (7)	0.093 (9)	0.079 (8)	0.027 (6)	0.032 (6)	0.037 (7)
C31	0.031 (9)	0.19 (3)	0.051 (10)	0.021 (12)	0.012 (7)	0.019 (14)
C32	0.054 (11)	0.16 (3)	0.055 (11)	−0.038 (15)	0.001 (8)	0.004 (14)
O33	0.045 (6)	0.095 (10)	0.075 (8)	−0.024 (6)	0.001 (5)	0.035 (7)
C34	0.111 (18)	0.077 (14)	0.096 (16)	−0.060 (13)	0.008 (14)	0.005 (12)
C35	0.125 (19)	0.064 (12)	0.091 (15)	−0.052 (13)	0.031 (14)	−0.018 (11)
O36	0.098 (10)	0.071 (8)	0.060 (7)	−0.047 (7)	0.027 (7)	−0.031 (6)
C37	0.137 (19)	0.041 (8)	0.039 (8)	−0.007 (10)	0.021 (10)	−0.013 (6)
C38	0.106 (15)	0.058 (10)	0.045 (9)	0.026 (10)	0.016 (9)	0.007 (7)
K1	0.0270 (12)	0.0395 (13)	0.0281 (11)	−0.0044 (10)	0.0020 (9)	−0.0003 (10)

### Geometric parameters (Å, °)

**Table:**

Pt1—N1	1.996 (8)	C23—H23A	0.9900
Pt1—N11	2.013 (8)	C23—H23B	0.9900
Pt1—Cl1	2.290 (3)	O24—C25	1.446 (19)
Pt1—Cl2	2.306 (3)	O24—K1	2.687 (9)
Cl2—K1	3.133 (4)	C25—C26	1.41 (3)
N1—N9	1.290 (12)	C25—K1	3.523 (16)
N1—C2	1.407 (13)	C25—H25A	0.9900
C2—C7	1.396 (13)	C25—H25B	0.9900
C2—C3	1.399 (15)	C26—O27	1.43 (3)
C3—C4	1.341 (16)	C26—K1	3.538 (19)
C3—H3	0.9500	C26—H26A	0.9900
C4—C5	1.492 (19)	C26—H26B	0.9900
C4—H4	0.9500	O27—C28	1.49 (2)
C5—C6	1.355 (18)	O27—K1	2.764 (10)
C5—H5	0.9500	C28—C29	1.48 (4)
C6—C7	1.421 (15)	C28—H28A	0.9900
C6—H6	0.9500	C28—H28B	0.9900
C7—N8	1.371 (14)	C29—O30	1.32 (3)
N8—O10	1.334 (11)	C29—H29A	0.9900
N8—N9	1.360 (12)	C29—H29B	0.9900
O10—K1^i^	2.897 (8)	O30—C31	1.47 (2)
N11—N19	1.329 (13)	O30—K1	2.785 (11)
N11—C12	1.374 (13)	C31—C32	1.46 (3)
C12—C17	1.371 (14)	C31—H31A	0.9900
C12—C13	1.420 (16)	C31—H31B	0.9900
C13—C14	1.366 (16)	C32—O33	1.33 (2)
C13—H13	0.9500	C32—H32A	0.9900
C14—C15	1.422 (17)	C32—H32B	0.9900
C14—H14	0.9500	O33—C34	1.44 (3)
C15—C16	1.346 (18)	O33—K1	2.804 (11)
C15—H15	0.9500	C34—C35	1.49 (3)
C16—C17	1.382 (14)	C34—H34A	0.9900
C16—H16	0.9500	C34—H34B	0.9900
C17—N18	1.398 (15)	C35—O36	1.41 (2)
N18—N19	1.331 (12)	C35—H35A	0.9900
N18—O20	1.336 (12)	C35—H35B	0.9900
O20—H20	0.8400	O36—C37	1.42 (2)
O21—C38	1.39 (2)	O36—K1	2.814 (11)
O21—C22	1.430 (18)	C37—C38	1.49 (3)
O21—K1	2.804 (10)	C37—H37A	0.9900
C22—C23	1.41 (2)	C37—H37B	0.9900
C22—H22A	0.9900	C38—H38A	0.9900
C22—H22B	0.9900	C38—H38B	0.9900
C23—O24	1.393 (19)	K1—O10^ii^	2.897 (8)
C23—K1	3.527 (15)		
			
N1—Pt1—N11	176.3 (3)	H28A—C28—H28B	108.4
N1—Pt1—Cl1	91.0 (3)	O30—C29—C28	109 (2)
N11—Pt1—Cl1	91.2 (3)	O30—C29—H29A	109.9
N1—Pt1—Cl2	89.0 (3)	C28—C29—H29A	109.9
N11—Pt1—Cl2	88.9 (3)	O30—C29—H29B	109.9
Cl1—Pt1—Cl2	177.77 (12)	C28—C29—H29B	109.9
Pt1—Cl2—K1	141.43 (13)	H29A—C29—H29B	108.3
N9—N1—C2	110.2 (8)	C29—O30—C31	113 (2)
N9—N1—Pt1	126.1 (7)	C29—O30—K1	115.1 (13)
C2—N1—Pt1	123.6 (7)	C31—O30—K1	112.2 (10)
C7—C2—C3	120.3 (10)	C32—C31—O30	112.2 (16)
C7—C2—N1	106.1 (9)	C32—C31—H31A	109.2
C3—C2—N1	133.5 (9)	O30—C31—H31A	109.2
C4—C3—C2	118.9 (11)	C32—C31—H31B	109.2
C4—C3—H3	120.5	O30—C31—H31B	109.2
C2—C3—H3	120.5	H31A—C31—H31B	107.9
C3—C4—C5	120.0 (11)	O33—C32—C31	111.7 (18)
C3—C4—H4	120.0	O33—C32—H32A	109.3
C5—C4—H4	120.0	C31—C32—H32A	109.3
C6—C5—C4	122.2 (11)	O33—C32—H32B	109.3
C6—C5—H5	118.9	C31—C32—H32B	109.3
C4—C5—H5	118.9	H32A—C32—H32B	108.0
C5—C6—C7	115.1 (11)	C32—O33—C34	110.8 (17)
C5—C6—H6	122.4	C32—O33—K1	117.9 (13)
C7—C6—H6	122.4	C34—O33—K1	113.7 (12)
N8—C7—C2	105.1 (9)	O33—C34—C35	109.2 (15)
N8—C7—C6	131.4 (9)	O33—C34—H34A	109.9
C2—C7—C6	123.4 (10)	C35—C34—H34A	109.9
O10—N8—N9	121.5 (9)	O33—C34—H34B	109.9
O10—N8—C7	128.0 (9)	C35—C34—H34B	109.9
N9—N8—C7	110.5 (8)	H34A—C34—H34B	108.3
N1—N9—N8	108.0 (8)	O36—C35—C34	108.2 (17)
N8—O10—K1^i^	126.6 (6)	O36—C35—H35A	110.1
N19—N11—C12	110.3 (8)	C34—C35—H35A	110.1
N19—N11—Pt1	119.5 (7)	O36—C35—H35B	110.1
C12—N11—Pt1	129.9 (7)	C34—C35—H35B	110.1
C17—C12—N11	107.9 (9)	H35A—C35—H35B	108.4
C17—C12—C13	120.7 (9)	C35—O36—C37	113.3 (13)
N11—C12—C13	131.4 (9)	C35—O36—K1	118.7 (12)
C14—C13—C12	115.5 (10)	C37—O36—K1	115.5 (9)
C14—C13—H13	122.3	O36—C37—C38	105.3 (12)
C12—C13—H13	122.3	O36—C37—H37A	110.7
C13—C14—C15	121.4 (12)	C38—C37—H37A	110.7
C13—C14—H14	119.3	O36—C37—H37B	110.7
C15—C14—H14	119.3	C38—C37—H37B	110.7
C16—C15—C14	123.5 (11)	H37A—C37—H37B	108.8
C16—C15—H15	118.3	O21—C38—C37	108.6 (14)
C14—C15—H15	118.3	O21—C38—H38A	110.0
C15—C16—C17	114.6 (11)	C37—C38—H38A	110.0
C15—C16—H16	122.7	O21—C38—H38B	110.0
C17—C16—H16	122.7	C37—C38—H38B	110.0
C12—C17—C16	124.3 (11)	H38A—C38—H38B	108.3
C12—C17—N18	103.8 (9)	O24—K1—O27	62.9 (3)
C16—C17—N18	131.9 (11)	O24—K1—O30	120.5 (4)
N19—N18—O20	119.6 (9)	O27—K1—O30	60.2 (4)
N19—N18—C17	111.6 (9)	O24—K1—O33	170.7 (3)
O20—N18—C17	128.7 (9)	O27—K1—O33	120.3 (4)
N11—N19—N18	106.3 (8)	O30—K1—O33	60.5 (4)
N18—O20—H20	109.5	O24—K1—O21	61.1 (3)
C38—O21—C22	111.3 (13)	O27—K1—O21	122.3 (4)
C38—O21—K1	116.5 (11)	O30—K1—O21	176.1 (4)
C22—O21—K1	109.8 (7)	O33—K1—O21	117.2 (4)
C23—C22—O21	110.4 (13)	O24—K1—O36	116.9 (4)
C23—C22—H22A	109.6	O27—K1—O36	176.5 (4)
O21—C22—H22A	109.6	O30—K1—O36	118.8 (4)
C23—C22—H22B	109.6	O33—K1—O36	59.4 (4)
O21—C22—H22B	109.6	O21—K1—O36	58.4 (3)
H22A—C22—H22B	108.1	O24—K1—O10^ii^	82.6 (3)
O24—C23—C22	110.2 (13)	O27—K1—O10^ii^	70.4 (3)
C22—C23—K1	79.6 (9)	O30—K1—O10^ii^	93.8 (3)
O24—C23—H23A	109.6	O33—K1—O10^ii^	106.7 (3)
C22—C23—H23A	109.6	O21—K1—O10^ii^	89.9 (3)
K1—C23—H23A	151.2	O36—K1—O10^ii^	113.1 (3)
O24—C23—H23B	109.6	O24—K1—Cl2	74.9 (2)
C22—C23—H23B	109.6	O27—K1—Cl2	90.0 (2)
K1—C23—H23B	93.2	O30—K1—Cl2	89.1 (2)
H23A—C23—H23B	108.1	O33—K1—Cl2	96.1 (2)
C23—O24—C25	115.9 (14)	O21—K1—Cl2	88.0 (2)
C23—O24—K1	116.0 (9)	O36—K1—Cl2	86.6 (3)
C25—O24—K1	113.5 (10)	O10^ii^—K1—Cl2	155.23 (19)
C26—C25—O24	112.3 (16)	O24—K1—C25	22.1 (4)
C26—C25—K1	79.1 (11)	O27—K1—C25	41.5 (5)
C26—C25—H25A	109.1	O30—K1—C25	101.2 (5)
O24—C25—H25A	109.1	O33—K1—C25	161.7 (5)
K1—C25—H25A	151.0	O21—K1—C25	81.1 (4)
C26—C25—H25B	109.1	O36—K1—C25	138.6 (5)
O24—C25—H25B	109.1	O10^ii^—K1—C25	71.7 (4)
K1—C25—H25B	94.7	Cl2—K1—C25	83.6 (4)
H25A—C25—H25B	107.9	O24—K1—C23	20.8 (4)
C25—C26—O27	111.0 (17)	O27—K1—C23	81.4 (4)
C25—C26—K1	77.9 (10)	O30—K1—C23	140.8 (5)
O27—C26—K1	46.8 (7)	O33—K1—C23	158.4 (5)
C25—C26—H26A	109.4	O21—K1—C23	41.2 (4)
O27—C26—H26A	109.4	O36—K1—C23	99.0 (4)
K1—C26—H26A	154.5	O10^ii^—K1—C23	79.2 (3)
C25—C26—H26B	109.4	Cl2—K1—C23	83.1 (3)
O27—C26—H26B	109.4	C25—K1—C23	39.9 (5)
K1—C26—H26B	91.4	O24—K1—C26	42.1 (5)
H26A—C26—H26B	108.0	O27—K1—C26	22.1 (5)
C26—O27—C28	115.1 (16)	O30—K1—C26	78.8 (5)
C26—O27—K1	111.1 (9)	O33—K1—C26	139.1 (5)
C28—O27—K1	113.7 (10)	O21—K1—C26	103.1 (5)
C29—C28—O27	108.3 (15)	O36—K1—C26	156.4 (5)
C29—C28—H28A	110.0	O10^ii^—K1—C26	78.8 (4)
O27—C28—H28A	110.0	Cl2—K1—C26	77.7 (4)
C29—C28—H28B	110.0	C25—K1—C26	23.1 (5)
O27—C28—H28B	110.0	C23—K1—C26	62.0 (5)
			
N1—Pt1—Cl2—K1	−113.6 (3)	C31—O30—K1—O33	−17.8 (13)
N11—Pt1—Cl2—K1	63.3 (3)	C29—O30—K1—O36	−161.1 (17)
Cl1—Pt1—N1—N9	59.4 (9)	C31—O30—K1—O36	−29.7 (14)
Cl2—Pt1—N1—N9	−118.4 (9)	C29—O30—K1—O10^ii^	−42.1 (17)
Cl1—Pt1—N1—C2	−116.4 (8)	C31—O30—K1—O10^ii^	89.3 (13)
Cl2—Pt1—N1—C2	65.8 (8)	C29—O30—K1—Cl2	113.3 (17)
N9—N1—C2—C7	1.6 (12)	C31—O30—K1—Cl2	−115.3 (13)
Pt1—N1—C2—C7	178.0 (7)	C29—O30—K1—C25	30.0 (18)
N9—N1—C2—C3	−176.5 (13)	C31—O30—K1—C25	161.4 (13)
Pt1—N1—C2—C3	−0.1 (17)	C29—O30—K1—C23	35.5 (19)
C7—C2—C3—C4	1.4 (18)	C31—O30—K1—C23	166.9 (13)
N1—C2—C3—C4	179.2 (12)	C29—O30—K1—C26	35.7 (17)
C2—C3—C4—C5	−2.3 (19)	C31—O30—K1—C26	167.1 (14)
C3—C4—C5—C6	3(2)	C32—O33—K1—O27	−20.5 (16)
C4—C5—C6—C7	−2.9 (18)	C34—O33—K1—O27	−152.6 (12)
C3—C2—C7—N8	176.0 (10)	C32—O33—K1—O30	−12.3 (15)
N1—C2—C7—N8	−2.4 (12)	C34—O33—K1—O30	−144.5 (13)
C3—C2—C7—C6	−1.2 (17)	C32—O33—K1—O21	164.0 (15)
N1—C2—C7—C6	−179.6 (10)	C34—O33—K1—O21	31.8 (13)
C5—C6—C7—N8	−174.4 (12)	C32—O33—K1—O36	155.5 (16)
C5—C6—C7—C2	2.0 (17)	C34—O33—K1—O36	23.4 (12)
C2—C7—N8—O10	−176.8 (10)	C32—O33—K1—O10^ii^	−97.2 (15)
C6—C7—N8—O10	0.1 (19)	C34—O33—K1—O10^ii^	130.7 (12)
C2—C7—N8—N9	2.5 (12)	C32—O33—K1—Cl2	73.1 (16)
C6—C7—N8—N9	179.4 (11)	C34—O33—K1—Cl2	−59.1 (12)
C2—N1—N9—N8	0.0 (12)	C32—O33—K1—C25	−15 (2)
Pt1—N1—N9—N8	−176.3 (7)	C34—O33—K1—C25	−147.0 (17)
O10—N8—N9—N1	177.7 (9)	C32—O33—K1—C23	159.7 (15)
C7—N8—N9—N1	−1.6 (12)	C34—O33—K1—C23	27.5 (17)
N9—N8—O10—K1^i^	147.6 (8)	C32—O33—K1—C26	−5.1 (18)
C7—N8—O10—K1^i^	−33.1 (15)	C34—O33—K1—C26	−137.2 (13)
Cl1—Pt1—N11—N19	65.1 (8)	C38—O21—K1—O24	145.4 (10)
Cl2—Pt1—N11—N19	−117.1 (8)	C22—O21—K1—O24	17.7 (9)
Cl1—Pt1—N11—C12	−121.8 (9)	C38—O21—K1—O27	160.4 (9)
Cl2—Pt1—N11—C12	55.9 (9)	C22—O21—K1—O27	32.6 (10)
N19—N11—C12—C17	2.0 (12)	C38—O21—K1—O33	−24.2 (10)
Pt1—N11—C12—C17	−171.5 (7)	C22—O21—K1—O33	−151.9 (9)
N19—N11—C12—C13	−178.6 (11)	C38—O21—K1—O36	−15.6 (10)
Pt1—N11—C12—C13	7.9 (17)	C22—O21—K1—O36	−143.4 (10)
C17—C12—C13—C14	0.7 (15)	C38—O21—K1—O10^ii^	−133.0 (10)
N11—C12—C13—C14	−178.6 (11)	C22—O21—K1—O10^ii^	99.2 (9)
C12—C13—C14—C15	0.3 (17)	C38—O21—K1—Cl2	71.6 (9)
C13—C14—C15—C16	0(2)	C22—O21—K1—Cl2	−56.1 (9)
C14—C15—C16—C17	−0.8 (18)	C38—O21—K1—C25	155.4 (10)
N11—C12—C17—C16	177.6 (10)	C22—O21—K1—C25	27.7 (10)
C13—C12—C17—C16	−1.9 (16)	C38—O21—K1—C23	153.4 (11)
N11—C12—C17—N18	−1.2 (11)	C22—O21—K1—C23	25.6 (9)
C13—C12—C17—N18	179.3 (9)	C38—O21—K1—C26	148.5 (10)
C15—C16—C17—C12	1.9 (17)	C22—O21—K1—C26	20.7 (10)
C15—C16—C17—N18	−179.7 (12)	C35—O36—K1—O24	−179.6 (12)
C12—C17—N18—N19	0.0 (12)	C37—O36—K1—O24	−40.0 (13)
C16—C17—N18—N19	−178.6 (11)	C35—O36—K1—O30	22.2 (14)
C12—C17—N18—O20	178.1 (10)	C37—O36—K1—O30	161.8 (11)
C16—C17—N18—O20	−1(2)	C35—O36—K1—O33	10.1 (12)
C12—N11—N19—N18	−2.0 (11)	C37—O36—K1—O33	149.7 (13)
Pt1—N11—N19—N18	172.4 (7)	C35—O36—K1—O21	−161.0 (14)
O20—N18—N19—N11	−177.0 (9)	C37—O36—K1—O21	−21.4 (11)
C17—N18—N19—N11	1.2 (12)	C35—O36—K1—O10^ii^	−86.3 (13)
C38—O21—C22—C23	179.7 (13)	C37—O36—K1—O10^ii^	53.3 (12)
K1—O21—C22—C23	−49.7 (13)	C35—O36—K1—Cl2	109.3 (13)
O21—C22—C23—O24	66.5 (15)	C37—O36—K1—Cl2	−111.2 (12)
O21—C22—C23—K1	35.5 (9)	C35—O36—K1—C25	−174.4 (13)
C22—C23—O24—C25	175.4 (14)	C37—O36—K1—C25	−34.9 (15)
K1—C23—O24—C25	−136.9 (14)	C35—O36—K1—C23	−168.3 (13)
C22—C23—O24—K1	−47.7 (14)	C37—O36—K1—C23	−28.8 (12)
C23—O24—C25—C26	−177.5 (16)	C35—O36—K1—C26	157.2 (15)
K1—O24—C25—C26	45 (2)	C37—O36—K1—C26	−63.3 (18)
C23—O24—C25—K1	137.9 (14)	Pt1—Cl2—K1—O24	−172.2 (3)
O24—C25—C26—O27	−64 (2)	Pt1—Cl2—K1—O27	126.0 (3)
K1—C25—C26—O27	−33.7 (13)	Pt1—Cl2—K1—O30	65.8 (4)
O24—C25—C26—K1	−30.0 (14)	Pt1—Cl2—K1—O33	5.6 (4)
C25—C26—O27—C28	179.1 (16)	Pt1—Cl2—K1—O21	−111.6 (3)
K1—C26—O27—C28	131.0 (14)	Pt1—Cl2—K1—O36	−53.2 (3)
C25—C26—O27—K1	48.1 (19)	Pt1—Cl2—K1—O10^ii^	162.9 (5)
C26—O27—C28—C29	−173.2 (17)	Pt1—Cl2—K1—C25	167.1 (4)
K1—O27—C28—C29	−43.5 (19)	Pt1—Cl2—K1—C23	−152.7 (4)
O27—C28—C29—O30	66 (2)	Pt1—Cl2—K1—C26	144.5 (5)
C28—C29—O30—C31	173.9 (14)	C26—C25—K1—O24	−139 (2)
C28—C29—O30—K1	−55 (2)	C26—C25—K1—O27	23.8 (11)
C29—O30—C31—C32	178.5 (17)	O24—C25—K1—O27	162.4 (17)
K1—O30—C31—C32	46.2 (18)	C26—C25—K1—O30	14.2 (14)
O30—C31—C32—O33	−59 (2)	O24—C25—K1—O30	152.8 (12)
C31—C32—O33—C34	174.4 (17)	C26—C25—K1—O33	16 (2)
C31—C32—O33—K1	41 (2)	O24—C25—K1—O33	155.1 (10)
C32—O33—C34—C35	170.2 (18)	C26—C25—K1—O21	−162.5 (14)
K1—O33—C34—C35	−54 (2)	O24—C25—K1—O21	−23.9 (12)
O33—C34—C35—O36	62 (2)	C26—C25—K1—O36	−150.9 (12)
C34—C35—O36—C37	179.2 (17)	O24—C25—K1—O36	−12.4 (17)
C34—C35—O36—K1	−40 (2)	C26—C25—K1—O10^ii^	104.6 (13)
C35—O36—C37—C38	−165.3 (16)	O24—C25—K1—O10^ii^	−116.8 (13)
K1—O36—C37—C38	53.0 (15)	C26—C25—K1—Cl2	−73.5 (13)
C22—O21—C38—C37	176.1 (12)	O24—C25—K1—Cl2	65.1 (12)
K1—O21—C38—C37	49.1 (15)	C26—C25—K1—C23	−160.4 (17)
O36—C37—C38—O21	−66.6 (17)	O24—C25—K1—C23	−21.8 (9)
C23—O24—K1—O27	−150.9 (10)	O24—C25—K1—C26	139 (2)
C25—O24—K1—O27	−13.0 (13)	C22—C23—K1—O24	135.1 (14)
C23—O24—K1—O30	−169.2 (9)	O24—C23—K1—O27	26.0 (9)
C25—O24—K1—O30	−31.3 (13)	C22—C23—K1—O27	161.1 (9)
C23—O24—K1—O21	14.9 (9)	O24—C23—K1—O30	14.8 (12)
C25—O24—K1—O21	152.8 (13)	C22—C23—K1—O30	149.9 (8)
C23—O24—K1—O36	33.0 (10)	O24—C23—K1—O33	−154.2 (9)
C25—O24—K1—O36	170.9 (13)	C22—C23—K1—O33	−19.0 (15)
C23—O24—K1—O10^ii^	−79.1 (9)	O24—C23—K1—O21	−160.0 (11)
C25—O24—K1—O10^ii^	58.7 (13)	C22—C23—K1—O21	−24.9 (8)
C23—O24—K1—Cl2	111.1 (9)	O24—C23—K1—O36	−150.5 (9)
C25—O24—K1—Cl2	−111.0 (13)	C22—C23—K1—O36	−15.4 (9)
C23—O24—K1—C25	−137.9 (17)	O24—C23—K1—O10^ii^	97.5 (9)
C25—O24—K1—C23	137.9 (17)	C22—C23—K1—O10^ii^	−127.4 (9)
C23—O24—K1—C26	−160.6 (12)	O24—C23—K1—Cl2	−65.1 (9)
C25—O24—K1—C26	−22.7 (13)	C22—C23—K1—Cl2	70.0 (8)
C26—O27—K1—O24	−17.5 (12)	O24—C23—K1—C25	23.2 (9)
C28—O27—K1—O24	−149.2 (13)	C22—C23—K1—C25	158.3 (12)
C26—O27—K1—O30	144.4 (13)	O24—C23—K1—C26	14.6 (9)
C28—O27—K1—O30	12.6 (12)	C22—C23—K1—C26	149.7 (10)
C26—O27—K1—O33	152.5 (12)	C25—C26—K1—O24	21.8 (11)
C28—O27—K1—O33	20.8 (14)	O27—C26—K1—O24	156.5 (16)
C26—O27—K1—O21	−32.2 (13)	C25—C26—K1—O27	−135 (2)
C28—O27—K1—O21	−164.0 (12)	C25—C26—K1—O30	−165.8 (14)
C26—O27—K1—O10^ii^	−109.1 (13)	O27—C26—K1—O30	−31.0 (12)
C28—O27—K1—O10^ii^	119.2 (13)	C25—C26—K1—O33	−172.2 (11)
C26—O27—K1—Cl2	55.4 (13)	O27—C26—K1—O33	−37.5 (16)
C28—O27—K1—Cl2	−76.3 (13)	C25—C26—K1—O21	17.8 (14)
C26—O27—K1—C25	−24.8 (13)	O27—C26—K1—O21	152.5 (12)
C28—O27—K1—C25	−156.6 (15)	C25—C26—K1—O36	53 (2)
C26—O27—K1—C23	−27.5 (13)	O27—C26—K1—O36	−171.9 (9)
C28—O27—K1—C23	−159.3 (13)	C25—C26—K1—O10^ii^	−69.5 (13)
C28—O27—K1—C26	−131.8 (18)	O27—C26—K1—O10^ii^	65.2 (12)
C29—O30—K1—O24	41.5 (18)	C25—C26—K1—Cl2	102.8 (13)
C31—O30—K1—O24	172.9 (13)	O27—C26—K1—Cl2	−122.5 (13)
C29—O30—K1—O27	22.7 (17)	O27—C26—K1—C25	135 (2)
C31—O30—K1—O27	154.1 (14)	C25—C26—K1—C23	14.1 (12)
C29—O30—K1—O33	−149.2 (18)	O27—C26—K1—C23	148.8 (14)

Symmetry codes: (i) *x*, −*y*+3/2, *z*+1/2; (ii) *x*, −*y*+3/2, *z*−1/2.

### Hydrogen-bond geometry (Å, °)

**Table:**

*D*—H···*A*	*D*—H	H···*A*	*D*···*A*	*D*—H···*A*
O20—H20···O10^iii^	0.84	1.76	2.466 (12)	141

Symmetry codes: (iii) *x*, −*y*+1/2, *z*−1/2.
